# Outcomes Among Undocumented Immigrant Kidney Transplant Recipients in California

**DOI:** 10.1001/jamanetworkopen.2022.54660

**Published:** 2023-02-13

**Authors:** Natsuki Eguchi, Ekamol Tantisattamo, Dean Chung, Uttam G. Reddy, Antoney Ferrey, Donald Dafoe, Hirohito Ichii

**Affiliations:** 1 Department of Surgery, University of California, Irvine; 2 Division of Nephrology and Hypertension, Department of Medicine, University of California, Irvine

## Abstract

**Importance:**

There are over 2 million undocumented immigrants (UI) in California, where currently, all individuals regardless of immigration status have access to kidney transplant. There is a medical perception that UI face a higher risk of transplant failure due to language barriers and lack of access to immunosuppressive medication and health care when compared with US residents (UR).

**Objective:**

To elucidate the kidney transplant outcomes of UI at an academic medical center in California.

**Design, Setting, and Participants:**

A retrospective cohort study was conducted from a single transplant center during an 8-year study period. Patients who received a kidney transplant at the University of California, Irvine, between January 1, 2012, and September 1, 2019, were included in this study. Data were analyzed from October 2020 to August 2021.

**Exposures:**

The primary exposure of this study was citizenship status. UI were defined as immigrants residing in the US without permission or legal documentation.

**Main Outcomes and Measures:**

The primary end point was all-cause graft loss defined as the return to dialysis, need for a second kidney transplant, or death. The secondary end points of this study were all-cause mortality and rejection. All-cause mortality between the 2 groups was compared using multiple Cox proportional hazard regression analysis. Other transplant outcomes, including all-cause graft loss and acute rejection, were examined by competing risks regressions with mortality and mortality plus graft loss serving as competing risks, respectively.

**Results:**

Of all 446 consecutive kidney transplant recipients, the mean (SD) age was 47 (13) years; 261 patients (59%) were male, and 114 (26%) were UI. During a median (IQR) follow-up time of 3.39 (0.04-8.11) years, 6 UI and 48 UR experienced all-cause graft loss. UR had a 192% (hazard ratio, 2.92; 95% CI, 1.21-6.85; *P* = .01) and 343% (hazard ratio, 4.34; 95% CI, 1.05-18.69; *P* = .04) significantly increased unadjusted risk for all-cause graft loss and all-cause mortality, respectively. These results became nonsignificant and were mostly attenuated when adjusted for age and ethnicity. Finally, there was no difference in incidence rate of kidney allograft rejection between the 2 groups (UR, 3.5 per 100 person-years vs UI, 2.4 per 100 person-years; rate ratio, 1.45; 95% CI, 0.90-5.05; *P* = .08).

**Conclusions and Relevance:**

This single-center cohort study found that kidney transplant outcomes of UI were not inferior to those of UR. Across the US, however, UI have consistently had unequal access to transplantation. These findings suggest that extending kidney transplants to UI is safe and does not portend worse outcomes. As a result, denying transplant according to immigration status not only results in higher costs but also worse end stage kidney disease outcomes for an already underserved population.

## Key Points

**Question:**

Are there any significant differences in kidney transplant outcomes between undocumented immigrants and US residents?

**Findings:**

This cohort study of 446 patients found that there was no difference in adjusted all-cause graft loss between undocumented immigrants and US residents.

**Meaning:**

These findings suggest that kidney transplant for undocumented immigrants is safe and should remain the treatment of choice for end-stage kidney disease for this underserved population.

## Teaser

This cohort study evaluates outcomes among undocumented immigrant kidney transplant recipients in California.

## Introduction

In 2014, there were an estimated 11 million undocumented immigrants (UI) living in the US, of which 2.6 million resided in California. Approximately 6500 of these UI have end-stage kidney disease (ESKD).^1^ Access to consistent dialysis for UI across the US is variable. Although all individuals are guaranteed dialysis in the event of an acute life-threatening condition through the Emergency Medical Treatment and Active Labor Act at Medicare-participating hospitals, outpatient maintenance dialysis is not covered for UI in most states. Medicare covers costs for maintenance dialysis and kidney transplant (KT) for US residents (UR), but not for UI. This is despite the fact that both maintenance dialysis and KT are substantially more cost-effective options and provide better outcomes for patients with ESKD compared with emergency-only dialysis. Therefore, access to maintenance dialysis and KT for UI is dependent upon state legislation.

California is one of the few states that use state funds to provide UI with access to maintenance dialysis and KT. A study that investigated the outcome of Medicaid-funded KT in UI receiving transplants in the US between 1999 and 2011 showed that of the 10 495 KT recipients, 346 were UI, of which the majority, 244 patients, received a transplant in California.^2^ Although this study had demonstrated that UI have a nonsignificantly lower risk for graft loss compared with UR (hazard ratio [HR], 0.67; *P* > .05) after adjustment for recipient and transplant characteristics, there still is an overbearing, long-held perception that UI have worse transplant outcomes due to financial and social issues. Kidney transplant outcome among UI in California, where there is the highest number of UI, has not been evaluated yet.^2^ With only 1 academic transplant center in Orange County, California, this single-center study at the University of California, Irvine, was conducted to compare KT outcomes of UI and UR to elucidate the association of residency status with KT outcomes.

## Methods

### Data Source and Study Population

This is a single-center cohort study of patients who received a KT at the University of California, Irvine, between January 1, 2012, and September 1, 2019. To minimize the impact of COVID-19 on our study, the follow-up date was set to March 1, 2020. To allow for a minimum follow-up time of 6 months, patients receiving KT after September 1, 2019, were excluded from this study. One patient who never regained consciousness after KT was excluded from the study. This study followed the Strengthening the Reporting of Observational Studies in Epidemiology (STROBE) reporting guideline and was approved by the University of California, Irvine, institutional review board. Because of the nature of the data collected, informed consent was waived by the institutional review board.

Each individual’s medical record was reviewed to retrieve information on race (Asian, Black, White, other [other included American Indian, Pacific Islander, and unknown]), ethnicity, citizenship status, donor type and characteristics, number of transplants, body mass index (calculated as weight in kilograms divided by height in meters squared), transplant characteristics, and pretransplant comorbidities including diabetes, cancer, hypertension, coronary artery disease, stroke, and heart failure. Race was self-reported and was included in this study as it is an important demographic factor that can influence graft outcome. Donor characteristics, including donor age, sex, race, ethnicity, Kidney Donor Profile Index (KDPI), presence of diabetes or hypertension, and birthplace, were gathered manually from the United Network for Organ Sharing. Hypertension was determined by ascertaining the diagnosis by health care practitioners during the pretransplant period or taking any antihypertensive medication for blood pressure control. Transplant characteristics included type of transplant, panel reactive antibody (PRA), Human Leukocyte Antigen (HLA) mismatch, cytomegalovirus infection risk, KDPI, and cold ischemic time. A zero-HLA mismatch (0MM) was based on a mismatch in the HLA-A, B, and DR loci.

### Exposure and End Points

The primary exposure of this study was citizenship status. UI were defined as immigrants residing in the US without permission or legal documentation. UR included both US citizens and permanent residents, as both are eligible for California State and federal Medicare funding for KT.

The primary end point was all-cause graft loss defined as the return to dialysis, need for a second KT, or death. The secondary end points were all-cause mortality and acute kidney allograft rejection determined by for-cause kidney allograft biopsy, which demonstrated acute cellular rejection, acute antibody-mediated rejection, or borderline changes.

### Statistical Analysis

Values are reported as mean and SD. We used the *t* test, χ^2^ test, and Fisher exact test to evaluate differences between UR and UI groups as appropriate. Kaplan-Meier analysis was used to examine the association of citizenship status with all-cause mortality, graft loss, and rejection. Cox proportional hazards regression analysis was used to examine the risk of all-cause mortality according to citizenship status. Survival analysis competing risk using Fine-Gray subdistribution model was used to examine the risks for all-cause graft loss and rejection with death and death plus graft loss as competing risks, respectively. Due to the low number of study outcomes, the Cox proportional hazard and cause-specific HRs were only adjusted with clinically relevant single variables, which included recipient characteristics (age, sex, race, ethnicity, dialysis duration, and pretransplant comorbidities including diabetes, stroke, hypertension, coronary artery disease, heart failure, cancer, dyslipidemia, and obesity), donor characteristics (age, sex, ethnicity, presence or absence of diabetes or hypertension, and terminal serum creatinine level), and transplant characteristics (type of transplant [living vs deceased donor KT (DDKT vs LDKT) and donation after brain death or cardiac death], cytomegalovirus serostatus, cold ischemia time, HLA mismatch, 0MM, induction immunosuppressive medications, panel-reactive antibody, and delayed graft function). Analyses were performed using R studio software version 1.4.1106 with the following packages: survival, ggplot2, ggpubr, survminer (R Project for Statistical Computing). Differences were considered statistically significant at *P* < .05. Data were analyzed from October 2020 to August 2021.

## Results

### Patient Demographics and Transplant Characteristics

Of the 446 patients who received a transplant at the University of California, Irvine, between January 1, 2012, and September 1, 2019, 114 patients were UI (Table 1). UI were more likely to be of younger age and less likely to have pretransplant diabetes compared with UR. Although half of the UR were Hispanic, up to 95% of UI were Hispanic. No difference was found in dialysis modality between the 2 groups; however, the UI group exhibited a significantly longer dialysis duration (mean [SD], 6.00 [0.37] in UR vs 7.10 [0.37] in UI; *P* = .04), and although 5% of patients in the UR group received preemptive transplants, no UI fell into this category.

**Table 1.** Patient Demographics and Transplant Characteristics^a^

Characteristics	Patients, No. (%)		*P* value
	US resident (n = 332)	Undocumented (n = 114)	
Patient age, mean (SD)	48.71 (14)	42.42 (11)	<.001
Body mass index, mean (SD)^b^	25.86 (0.32)	25.73 (0.42)	.79
Recipient sex			
Female	138 (41.6)	47 (41.2)	.99
Male	194 (58.4)	67 (58.8)	
Recipient race			
Asian	75 (22.6)	4 (3.5)	<.001
Black	13 (3.9)	0 (0)	
White	218 (65.7)	88 (77.2)	
Other/unknown^c^	26 (2.4)	22 (19.3)	
Recipient ethnicity			
Hispanic	171 (15.7)	107 (93.4)	<.001
Pretransplant comorbidities			
Diabetes	116 (34.9)	24 (21.1)	.008
Coronary artery disease	63 (19.0)	17 (14.9)	.40
Stroke	26 (7.8)	4 (3.5)	.17
Hypertension	306 (92.2)	110 (96.5)	.17
Heart failure	26 (7.8)	11 (9.6)	.68
Dyslipidemia	80 (24.1)	19 (16.7)	.13
Dialysis history			
Dialysis	317 (95.4)	114 (100)	.027
Dialysis duration, mean (SD)	6.00 (0.37)	7.10 (0.37)	.04
Dialysis modality			
Hemodialysis	263 (79.2)	102 (89.5)	.33
Peritoneal	35 (10.5)	8 (7.0)	
Both	16 (4.8)	4 (3.5)	
Type of transplant			
Living donor transplant	98 (29.5)	20 (17.5)	.017
Donation after cardiac death	51 (15.4)	16 (14.0)	.41
PRA			
Low (0%-19%)	237 (71.4)	82 (71.9)	.99
Medium (20%-79%)	39 (11.7)	13 (11.4)	
High (80%-100%)	56 (16.8)	19 (16.7)	
Human leukocyte antigen mismatch	3.70 (0.10)	3.45 (0.18)	.21
Zero mismatch	27 (8.1)	17 (14.9)	.16
Cytomegalovirus high risk	55 (16.6)	9 (7.9)	.06
KDPI (deceased donor kidney transplant only), mean (SD)	51.85 (1.67)	47.37 (2.75)	.17
Cold ischemic time, mean (SD)	14.32 (0.52)	17.22 (0.91)	.005

Abbreviations: KDPI, Kidney Donor Profile Index; PRA, panel reactive antibody.

^a^ Pretransplant recipient characteristics and transplant characteristics of KT between 2012-2019.

^b^ Body mass index is calculated as weight in kilograms divided by height in meters squared.

^c^ Other includes American Indian, Pacific Islander, and unknown.

UI were less likely to undergo LDKT (30% in UR vs 18% in UI; χ^2^_1_ = 5.65; *P* = .02) (Table 1). Deceased donor kidneys of the UI group had longer cold ischemic times, but there was no difference in KDPI between the 2 groups. PRA profiles of UR and UI did not differ significantly; however, although the rate of transplant for UI was 15% compared with 8% in UR, this difference was not statistically significant (χ^2^_1_ = 1.94; *P* = .16). In addition, UI had an only 8% rate of high risk for cytomegalovirus, compared with 17% among UR, but this difference was not statistically significant (χ^2^_2_ = 5.61; *P* = .06).

### Association of Citizenship With Transplant Outcomes

During a median (IQR) follow-up time of 3.39 (0.04-8.11) years, 48 UR and 6 UI experienced all-cause graft loss (Table 2). Within the 48 graft losses in the UR group, 24 were identified as dialysis after graft loss (DAGL); meanwhile, 4 of 6 graft losses were DAGL in the UI group. Graft survival at 8 years posttransplantation was 86% in UR and 95% in the UI group (Table 2). In terms of all-cause mortality, during the 8-year follow-up, 26 UR and 2 UI patients died, with an 8-year survival rate of 92% and 98%, respectively. Finally, 36 UR and 9 UI patients experienced biopsy-proven rejection during the follow-up period.

**Table 2.** Primary and Secondary Outcomes^a^

Outcomes	Patients, No. (%)		*P* value
	US resident (n = 332)	Undocumented (n = 113)	
All-cause graft loss			
Total No. of events	48 (14.5)	6 (5.3)	NA
Death with functioning graft	25 (52.1)	2 (33.3)	NA
Dialysis after graft loss	23 (47.9)	4 (66.7)	NA
Survival rate (8 y), %	86	95	NA
Total follow-up time, person-years	1078.28	362.30	NA
Incident rate per 100 person-years	4.4	1.7	.02
Dialysis after graft loss			
No. of events	24 (7.2)	4 (3.5)	NA
Survival rate (8 y)	93	97	NA
Total follow-up time (person-years)	1078.28	362.30	NA
Incident rate (per 100 person-years)	2.2	1.1	.19
All-cause mortality			
No. of events	26 (7.8)	2 (1.8)	NA
Survival rate (8 y)	92	98	NA
Total follow-up time (person-years)	1113.50	369.33	NA
Incident rate (per 100 person-years)	2.3	0.5	.02
Rejection			
No. of events	36 (10.8)	9 (8.0)	NA
Survival rate (8 y)	89	92	NA
Total follow-up time (person-years)	1010.57	358.59	NA
Incident rate (per 100 person-years)	3.5	2.5	.08

Abbreviation: NA, not applicable.

^a^ Number of events and incidence rates for all study outcomes.

In unadjusted results, UR had a 192% increased risk for all-cause graft loss compared with UI (HR, 2.92; 95% CI, 1.25-6.85; *P* = .01) (Figure and Table 3). The results were slightly attenuated but remained significant when adjusted with recipient demographics, comorbidities, and/or transplant characteristics (eTable 1 in Supplement 1). When stratified for DDKT, UR still had a significant 184% increased unadjusted risk for all-cause graft loss (HR, 2.84; 95% CI, 1.11-7.22; *P* = .02), which became nonsignificant when adjusted for age (HR, 2.53; 95% CI, 0.97-6.59; *P* = .06), ethnicity (HR, 2.23; 95% CI, 0.84-6.93; *P* = .11), or delayed graft function (HR, 2.28; 95% CI, 0.89-5.85; *P* = .09) (Table 4 and eTable 2 in Supplement 1). Although there was no significant difference in unadjusted DAGL (UR HR, 2.27; 95% CI, 0.78-6.56; *P* = .13) between the 2 groups, UR had a 343% increased risk for all-cause mortality (HR, 4.43; 95% CI, 1.05-18.69; *P* = .04) (Figure and Table 3 and eTable 3 and eTable 4 in Supplement 1). The HR became nonsignificant after stratifying for DDKT (UR HR, 4.21; 95% CI, 0.99-17.91; *P* = .05). In both cases (all transplants and DDKT only), the increased risk for all-cause mortality was attenuated and became nonsignificant when adjusted for recipient demographics, comorbidities, or transplant characteristics (eTable 5 and eTable 6 in Supplement 1). Interestingly, in DDKT, the risk for all-cause mortality was not associated with ethnicity (UR HR, 2.66; 95% CI, 0.58-12.14; *P* = .21) and donation after cardiac death (UR HR, 1.85; 95% CI, 0.53-6.44; *P* = .33) (Table 4). There was no difference in incidence rate of kidney allograft rejection between the 2 groups (UR, 3.5 per 100 person-years vs UI, 2.4 per 100 person-years; *P* = .08). Lastly, no significant difference was found in unadjusted risk for rejection for both all transplants (UR HR, 1.44; 95% CI, 0.53-6.44; *P* = .33) and DDKT (UR HR, 1.06; 95% CI, 0.47-2.37; *P* = .90) (Figure and Table 3 and eTable 7 and eTable 8 in Supplement 1).

**Figure.**
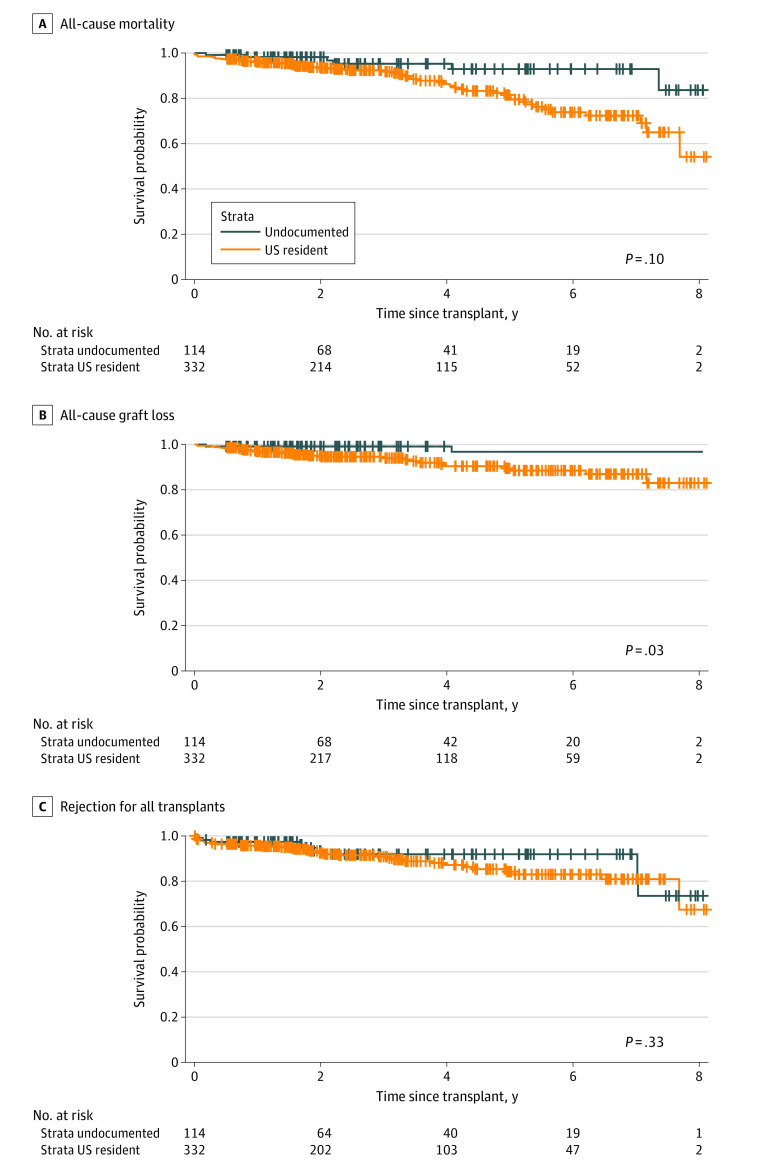
Kaplan-Meier Curve for All-Cause Mortality, All-Cause Graft Loss, and Rejection for All Transplants

**Table 3.** Unadjusted and Adjusted Survival Rates for All Transplants^a^

Model	All-cause graft loss		DAGL		All-cause mortality		Rejection	
	HR (95% CI)	*P* value	HR (95% CI)	*P* value	HR (95% CI)	*P* value	HR (95% CI)	*P* value
Unadjusted	2.92 (1.25-6.85)	.01	2.27 (0.78-6.56)	.13	4.43 (1.05-18.69)	.04	1.44 (0.69-2.99)	.33
Adjusted								
Demographics								
Age	2.45 (1.02-5.9)	.04	2.52 (0.84-7.5)	.10	2.57 (0.59-11.23)	.21	1.67 (0.79-3.53)	.18
Race	2.6 (1.1-6.15)	.03	2.94 (0.95-9.07)	.06	3.62 (0.28-15.78)	.08	1.73 (0.81-3.71)	.16
Ethnicity	2.7 (1.11-6.53)	.03	2.52 (0.84-7.53)	.10	3.28 (0.74-14.54)	.12	1.85 (0.87-3.91)	.11
Comorbidities								
Coronary artery disease	2.98 (1.27-7.00)	.01	2.31 (0.8-6.7)	.12	4.51 (1.07-19.03)	.04	1.44 (0.69-2.99)	.33
Diabetes	2.74 (1.16-6.48)	.02	2.24 (0.76-6.56)	.14	3.87 (0.91-16.43)	.07	1.45 (0.69-3.03)	.33
Obesity	2.79 (1.19-6.55)	.02	2.16 (0.75-6.26)	.16	4.43 (1.03-18.36)	.045	1.42 (0.68-2.95)	.35
Transplant								
Zero mismatch	2.9 (1.23-6.83)	.01	2.22 (0.78-6.58)	.14	4.41 (1.04-18.69)	.04	1.46 (0.7-3.05)	.32
Delayed graft function	2.52 (1.26-6.91)	.03	1.78 (0.61-5.23)	.29	4.10 (0.97-17.32)	.06	1.34 (0.64-2.79)	.44

Abbreviations: DAGL, dialysis after graft loss; HR, hazard ratio.

^a^ Unadjusted and adjusted hazards ratio for all-cause graft loss, DAGL, all-cause mortality, and rejection for all transplants.

**Table 4.** Unadjusted and Adjusted Survival Rates for Deceased Donor Kidney Transplant^a^

Model	All-cause graft loss		DAGL		All-cause mortality		Rejection	
	HR (95% CI)	*P* value	HR (95% CI)	*P* value	HR (95% CI)	*P* value	HR (95% CI)	*P* value
Unadjusted	2.84 (1.11-7.22)	.02	2.13 (0.62-7.29)	.23	4.21 (0.99-17.91)	.05	1.06 (0.47-2.37)	.90
Adjusted								
Demographics								
Age	2.53 (0.97-6.59)	.06	2.33 (0.67-8.16)	.19	2.87 (0.65-12.62)	.16	1.22 (0.53-2.77)	.64
Ethnicity	2.23 (0.84-5.93)	.11	2.06 (0.58-7.34)	.27	2.66 (0.58-12.14)	.21	1.23 (0.54-2.82)	.62
Race	2.94 (0.95-9.07)	.06	2.23 (0.62-8.12)	.22	3.46 (0.79-15.21)	.10	1.14 (0.49-2.66)	.77
Comorbidities								
Coronary artery disease	3.13 (1.22-8.00)	.02	2.4 (0.7-8.27)	.17	5.59 (1.07-19.65)	.04	1.05 (0.47-2.36)	.91
DM	2.71 (1.05-6.96)	.04	2.11 (0.61-7.34)	.34	3.75 (0.87-16.11)	.08	1.05 (0.47-2.39)	.90
Obesity	2.83 (1.11-7.21)	.03	2.16 (0.63-7.40)	.22	4.15 (0.97-17.69)	.05	1.05 (0.47-2.36)	.91
Transplant								
Delayed graft function	2.28 (0.89-5.85)	.09	1.47 (0.42-5.14)	.55	3.68 (0.89-16.24)	.007	0.89 (0.39-2.03)	.78
Zero mismatch	2.73 (1.06-6.99)	.04	2.08 (0.6-7.23)	.25	3.98 (0.93-17)	.06	1.07 (0.47-2.42)	.88

Abbreviations: DAGL, dialysis after graft loss; HR, hazard ratio.

^a^ Unadjusted and adjusted hazards ratio for all-cause graft loss, DAGL, all-cause mortality, and rejection for deceased donor kidney transplant.

## Discussion

This cohort study compared KT outcomes of UI and UR who received transplants at the University of California, Irvine, between 2012 and 2019. UR had a significantly increased unadjusted risk for all-cause graft loss (192%) and all-cause mortality (343%). These results became nonsignificant and were mostly attenuated when adjusted for age and ethnicity, suggesting that the lower age and Hispanic ethnicity more frequent in the UI group were the primary drivers of reduced mortality. There were no significant differences in DAGL and rejection between the 2 groups.

The findings from our study are partly in line with previously reported results that demonstrated that UI had a lower risk for transplant graft loss compared with UR. The study by Shen et al,^2^ which examined Medicaid-insured KT outcomes in adult nonresident aliens in the US between 1990 and 2011, found that of the 278 779 adult patients, 346 patients were UI, and that nonresident aliens had a less than 45% lower unadjusted risk for all-cause graft loss (HR, 0.48; 95% CI, 0.35-0.65), DAGL (HR, 0.55; 95% CI, 0.39-0.79), and death compared with US citizens at 5 years (HR, 0.42; 95% CI, 0.26-0.67). Limiting the inclusion criteria to only Medicaid patients likely results in the representation of only a subgroup of UI who have stronger family and financial support and are able to better navigate the health care system, thus limiting the generalizability of the study. Similarly, a single-center study^3^ of 289 pediatric KT recipients in California found that even after adjusting for recipient, donor, and transplant characteristics, UI children had a 62% lower adjusted risk for transplant loss compared with permanent residents and US citizens at 5 years (HR, 0.38; 95% CI, 0.15-0.96). Furthermore, most recently, a single center study^4^ at the University of California, Davis reported that UI had 85.9% and 100% graft survival at 8 years for deceased and living donor transplants, respectively. All in all, these studies, along with ours, call into question the long-held belief that undocumented immigrants have worse transplant outcomes.

There are a few explanations for the findings of our study. First, the better outcomes of the UI group may largely be explained by the younger age and the lower prevalence of diabetes, both of which were associated with the HR of all-cause mortality. In addition, considering that the UI group still had better outcomes, although not significant, when stratified for deceased donor kidney transplant, this may suggest that recipient characteristics play a more important role than donor kidney condition in determining KT outcomes. Thus, the younger age and lower prevalence of diabetes likely played a major role in the improved outcomes in the UI group. Importantly, these findings are in line with the nationwide study by Shen et al^2^ suggesting that UI patients that receive KT are generally younger and healthier. Second, in addition to the younger age, UI patients who are able to undergo KT may represent a subset of UI that have stronger family and financial support to be able to navigate the health care system, thus having a more robust socioeconomic support system. Future mixed method studies with a qualitative part may explore these confounders to explain further the results of our current study. Lastly, although the residency status of deceased donors is not available, the higher prevalence of 0MM transplantation in the UI group may suggest better HLA matching in the UI group. This may suggest some representation of UI in the deceased donor pool as it has been established that certain HLA haplotypes are found in higher frequency in certain populations.^5^ This is supported in the study by Shen et al,^2^ which found that while only 206 UI underwent DDKT during the 20 years, 990 deceased donors were UI. In summary, the better outcomes of UI in our study may be explained by selection bias for UI with younger age and lower prevalence of diabetes, as well as a better donor-recipient match compared with UR.

According to the Emergency Medical Treatment and Labor Act of 1985, UI in every state are eligible for emergency dialysis in the event of an acute life-threatening condition.^6^ Nevertheless, UI in most states are not eligible for maintenance dialysis as patients are not in imminent death, and only 12 states, including California, New York, and Illinois, have established Medicaid programs that pay for maintenance dialysis of UI.^7^ In California, maintenance dialysis is considered a life-sustaining medical treatment covered by the California Medical Assistance Program (Medi-Cal).^8^ Furthermore, in California, UI with ESKD are eligible for coverage by Medi-Cal when receiving KT; therefore, all individuals in California theoretically have equal access to KT regardless of immigration status. Thus, the noninferior transplant outcomes of UI in comparison with UR that were identified in our study can be partially attributed to the state’s provision of insurance support for UI receiving KT and maintenance dialysis.

Granting maintenance dialysis and KT access to all ESKD patients is not only essential to provide ESKD patients with better outcomes, but to also reduce the cost of ESKD treatment. In 2019, the total Medicare spending on the treatment of ESKD patients totaled $35.9 billion, accounting for 7.2% of the overall Medicare-paid claims in the fee-for-service system.^9^ Nguyen et al^10^ previously demonstrated that there is a total savings of $5768 per person per month when switching from emergency dialysis to maintenance dialysis, likely a result of decreased occurrence and duration of emergency department visits and hospitalization. The savings are even greater when a patient undergoes KT. Illinois reports that for every patient who receives state-funded maintenance dialysis for over 2.7 years, the state would have saved money if the patient had undergone KT instead.^11^ Supporting this study, Linden et al^12^ state that with an estimated 8-year life expectancy of a KT, there is a net savings of $321 000 per patient when a patient undergoes KT. Thus, individual states and the nation as a whole would save substantial taxpayer dollars by granting undocumented immigrants access to KT. Despite these benefits, in most states, UI are still denied these better ESKD treatment options. The reasons are unclear but likely arise from several factors contributing to the perception regarding worse transplant outcomes. In addition, social determinants of health may lead to this disparity to transplant access.^13^

Although maintenance dialysis and eventual KT are the standard of care for patients with ESKD, immunosuppressants, commonly consisting of tacrolimus with or without steroids, are also a sine qua non for maintaining the kidney health of patients following KT.^14^ Nevertheless, posttransplant outpatient drugs, including immunosuppressants, cost up to $31 900 without medical insurance, which is a daunting financial burden for UI who are significantly more likely to be uninsured than URs.^15,16,17^ To this day, the enigma of immunosuppressant coverage for UIs during the posttransplant period remains unresolved. Some states, through state-level legislation, provide viable solutions that enhance the affordability of immunosuppressants for UI following a transplant. Several states, namely Alaska, California, New Mexico, and New York, offer medical assistance programs to UI who have permanently resided under color of law (PRUCOL) status by using federal and state-exclusive funds.^18^ For instance, UI with PRUCOL status in California are eligible for full-scope Medi-Cal which covers immunosuppressants, such as tacrolimus, mycophenolic acid, and prednisone, if they meet other eligibility criteria.^8^ On a separate note, there have been several notable legal reformations in the past decade that seek to expand medical insurance coverage for UI. California recently passed Senate Bill 104 (SB104) that would extend eligibility for full-scope Medi-Cal benefits to undocumented youth who are aged 19 to 25 years regardless of PRUCOL status.^19^ This is a landmark victory for undocumented pediatric KT, considering that this is one major obstacle against favorable graft outcome for this patient population after they turn 21 years of age.^3^

### Limitations and Strengths

There are several limitations to our study. The first is that since this is a single-center study, it has a small sample size and low prevalence of study outcome; therefore, our study lacks statistical power, and HRs were only able to be adjusted with a single variable. Additionally, rejection biopsies in this study were all conducted to evaluate graft dysfunction rather than for surveillance. As a result, the number of patients with rejection was extremely low, adding to the lack of statistical power. Second, there may be several residual confounding factors such as genetic factors, environmental factors, and psychosocial factors that may have affected transplant outcomes that we are unable to ascertain and include in our study (eFigure 2 in Supplement 1). Third, there may have been a misclassification of undocumented immigrants as UR. If genetic factors were to have played a role in our results, our study would not have accounted for that. In addition, there is selection bias considering that UI who are able to undergo KT are more likely to have stronger family and financial support as well as being younger and more capable of navigating the health care system and thus do not represent the UI population as a whole; thus our study lacks generalizability. Despite this, the UCI transplant program does not distinguish between UIs and URs during the selection process; instead both groups are subjected to the same rigorous multidisciplinary evaluation. Our team includes a licensed social worker and financial coordinator among the other clinical specialists, who help to stratify our patients’ individual, financial, and social risk for success posttransplant. Qualitative studies as part of mixed method studies will provide further explanation of our study results.

The strength of our study is that there were a low number of missing data, and since the data were gathered manually in a consistent fashion, our study has increased accuracy. Finally, since this is a single-center study, all patients were treated by the same transplant clinicians, which increases consistency throughout the entire process, including pretransplant evaluation, donor selection, recipient selection, immunosuppression, and posttransplant management.

## Conclusions

No significant difference in mortality, DAGL, and rejection were evident among the UR and UI groups. However, the UI group did exhibit an insignificant reduced risk for all-cause mortality which was likely due to their younger age. In conclusion, the KT outcomes of the UI are not inferior to those of the UR; however, the UI have long been a minority in KT in the US. Extending KT to UI may be a reasonable option to offer better ESKD outcomes to this underserved population.

## Notes

Supplement 1. **eTable 1. **All-Cause Graft Loss for All Transplant (LDRT and DDRT) **eTable 2. **All-Cause Graft Loss for DDRT Only **eTable 3. **Dialysis After Graft Loss (DAGL) for All Transplant **eTable 4. **DAGL for DDRT Only **eTable 5. **All-Cause Mortality for All Transplants **eTable 6. **All-Cause Mortality for DDRT Only **eTable 7. **Rejection for All Transplant **eTable 8. **Rejection for DDRT Only **eFigure 1. **Cumulative Incidence Curve for A) Death Censored Graft Loss B) Death and Graft Loss Censored Rejection by Residency Status **eFigure 2. **Conceptual Framework

Supplement 2.
Data Sharing Statement

## References

[1] Raghavan R. Caring for undocumented immigrants with kidney disease. Am J Kidney Dis. 2018;71(4):488-494. doi:10.1053/j.ajkd.2017.09.011 29198642

[2] Shen JI, Hercz D, Barba LM, . Association of citizenship status with kidney transplantation in medicaid patients. Am J Kidney Dis. 2018;71(2):182-190. doi:10.1053/j.ajkd.2017.08.014 29128413 PMC5794566

[3] McEnhill ME, Brennan JL, Winnicki E, . Effect of immigration status on outcomes in pediatric kidney transplant recipients. Am J Transplant. 2016;16(6):1827-1833. doi:10.1111/ajt.13683 26699829

[4] Luce MS, Kleber KT, Abdallah AC, Basmaci UN, Perez RV, Troppmann C. Outcomes of kidney transplant in undocumented immigrants. JAMA Surg. 2021;156(11):1063-1064. doi:10.1001/jamasurg.2021.2870 34319374 PMC8319817

[5] Shankarkumar U. Complexities and similarities of HLA antigen distribution in Asian subcontinent. Indian J Hum Genet. 2010;16(3):108-110. doi:10.4103/0971-6866.73397 21206696 PMC3009419

[6] Zibulewsky J. The Emergency Medical Treatment and Active Labor Act (EMTALA): what it is and what it means for physicians. Proc (Bayl Univ Med Cent). 2001;14(4):339-346. doi:10.1080/08998280.2001.11927785 16369643 PMC1305897

[7] Cervantes L, Fischer S, Berlinger N, . The illness experience of undocumented immigrants with end-stage renal disease. JAMA Intern Med. 2017;177(4):529-535. doi:10.1001/jamainternmed.2016.8865 28166331

[8] Department of Health Care Services. Medi-Cal Eligibility Procedures Manual. 2021. Accessed January 9, 2023. https://www.dhcs.ca.gov/services/medi-cal/eligibility/Pages/MedEligProcManual.aspx

[9] Saran R, Robinson B, Abbott KC, . US renal data system 2019 annual data report: epidemiology of kidney disease in the United States. Am J Kidney Dis. 2020;75(1)(suppl 1):A6-A7. doi:10.1053/j.ajkd.2019.09.003 31704083

[10] Nguyen OK, Vazquez MA, Charles L, . Association of scheduled vs emergency-only dialysis with health outcomes and costs in undocumented immigrants with end-stage renal disease. JAMA Intern Med. 2019;179(2):175-183. doi:10.1001/jamainternmed.2018.5866 30575859 PMC6439652

[11] Rizzolo K, Cervantes L. Barriers and solutions to kidney transplantation for the undocumented latinx community with kidney failure. Clin J Am Soc Nephrol. 2021;16(10):1587-1589. doi:10.2215/CJN.03900321 34556499 PMC8499002

[12] Linden EA, Cano J, Coritsidis GN. Kidney transplantation in undocumented immigrants with ESRD: a policy whose time has come? Am J Kidney Dis. 2012;60(3):354-359. doi:10.1053/j.ajkd.2012.05.016 22784995

[13] Wesselman H, Ford CG, Leyva Y, . Social determinants of health and race disparities in kidney transplant. Clin J Am Soc Nephrol. 2021;16(2):262-274. doi:10.2215/CJN.04860420 33509963 PMC7863655

[14] Kalluri HV, Hardinger KL. Current state of renal transplant immunosuppression: present and future. World J Transplant. 2012;2(4):51-68. doi:10.5500/wjt.v2.i4.51 24175197 PMC3782235

[15] Bentley T. 2017 U.S. organ and tissue transplant cost estimates and discussion. 2017. Accessed October 2021. https://costprojections.com/wp-content/uploads/2019/03/milliman.2017.pdf

[16] Foundation KF. Health Coverage of Immigrants. 2022. Accessed October, 2021. https://www.kff.org/racial-equity-and-health-policy/fact-sheet/health-coverage-of-immigrants/

[17] Grubbs V. Undocumented immigrants and kidney transplant: costs and controversy. Health Aff (Millwood). 2014;33(2):332-335. doi:10.1377/hlthaff.2013.0462 24493776 PMC4062914

[18] Center NIL. Medical assistance programs for immigrants in various states. 2023. Accessed October, 2021. https://www.nilc.org/issues/health-care/medical-assistance-various-states/

[19] Bill Track 50. CA SB104. 2019. Accessed January 13, 2023. https://www.billtrack50.com/BillDetail/1012077

