# Supplementary material for: Outcomes Among Undocumented Immigrant Kidney Transplant Recipients in California
Source: JAMA Netw Open. 2023 Feb 13;6(2):e2254660. doi: 10.1001/jamanetworkopen.2022.54660 (PMC9926318; doi:10.1001/jamanetworkopen.2022.54660)
Supplement: Supplement 1. — eTable 1. All-Cause Graft Loss for All Transplant (LDRT and DDRT) eTable 2. All-Cause Graft Loss for DDRT Only eTable 3. Dialysis After Graft Loss (DAGL) for All Transplant eTable 4. DAGL for DDRT Only eTable 5. All-Cause Mortality for All Transplants eTable 6. All-Cause Mortality for DDRT Only eTable 7. Rejection for All Transplant eTable 8. Rejection for DDRT Only eFigure 1. Cumulative Incidence Curve for A) Death Censored Graft Loss B) Death and Graft Loss Censored Rejection by Residency Status eFigure 2. Conceptual Framework [file jamanetwopen-e2254660-s001.pdf]

Supplemental Online Content

Eguchi N, Tantisattamo E, Chung D, et al. Outcomes among undocumented immigrant kidney transplant recipients in California. *JAMA Netw Open*. 2023;6(2):e2254660. doi:10.1001/jamanetworkopen.2022.54660

eTable 1. All-Cause Graft Loss for All Transplant (LDRT and DDRT)
eTable 2. All-Cause Graft Loss for DDRT Only
eTable 3. Dialysis After Graft Loss (DAGL) for All Transplant
eTable 4. DAGL for DDRT Only
eTable 5. All-Cause Mortality for All Transplants
eTable 6. All-Cause Mortality for DDRT Only
eTable 7. Rejection for All Transplant
eTable 8. Rejection for DDRT Only
eFigure 1. Cumulative Incidence Curve for A) Death Censored Graft Loss B) Death and Graft Loss Censored Rejection by Residency Status
eFigure 2. Conceptual Framework

This supplemental material has been provided by the authors to give readers additional information about their work.

© 2023 Eguchi N et al. *JAMA Network Open.*

eTable 1. All-Cause Graft Loss for All Transplant (LDRT and DDRT)

	Hazards ratio (US resident vs. Undocumented Immigrants)			
Unadjusted	Unadjusted	HR*	95% CI	P
		2.92	[1.25,6.85]	0.01
Adjusted	Adjusted variables	HR*	95% CI	P
	**Demographics**			
	Age	2.45	[1.02,5.9]	0.04
	Race (AA)	2.6	[1.1,6.15]	0.03
	Ethnicity	2.7	[1.11,6.53]	0.03
	Obesity	2.79	[1.19,6.55]	0.02
	**Comorbidities**			
	CAD	2.98	[1.27,7.00]	0.01
	DM	2.74	[1.16,6.48]	0.02
	HTN	2.89	[1.23,6.77]	0.01
	Stroke	2.83	[1.2,6.65]	0.02
	HF	2.93	[0.01,0.88]	0.01
	Cancer	2.83	[1.2,6.65]	0.02
	**Transplant Characteristics**			
	CIT (/5 hour)	3.04	[1.3,7.11]	0.01
	CMV High risk	2.85	[1.21,6.69]	0.02
	Dialysis duration	2.92	[1.25,6.85]	0.01
	DGF	2.52	[1.07,5.92]	0.03
	High PRA	2.95	[1.26,6.91]	0.01
	HLA	2.87	[1.22,6.73]	0.02
	Living Donor	2.99	[1.28,7.00]	0.01
	Simulect Induction	2.98	[1.27,7.00]	0.01
	0MM	2.9	[1.23,6.83]	0.01
	**Donor Characteristics**			
	Age	2.87	[1.23,6.74]	0.02
	Dm	3.01	[1.28,7.08]	0.01
	Ethnicity	2.95	[1.25,6.97]	0.01
	Gender	2.94	[1.25,6.90]	0.01
	HTN	3.17	[1.35,7.46]	0.01

© 2023 Eguchi N et al. *JAMA Network Open.*

	Serum Cr	2.93	[1.25,6.88]	0.01

0MM, 0-human leukocyte antigen mismatch; AA, African American; CAD, coronary artery disease; CI, Confidence interval; CIT, cold ischemic time; CMV, cytomegalovirus; Cr, creatinine; DDKT, deceased donor kidney transplant; DGF, delayed graft function; DM, diabetes mellitus; HF, heart failure; HLA, human leukocyte antigen; HR, hazard ratio; HTN, hypertension; LDKT, living donor kidney transplant; PRA, panel reactive antibody; UI, undocumented immigrants; UR, US residents
* UI is a reference

© 2023 Eguchi N et al. *JAMA Network Open.*

eTable 2. All-Cause Graft Loss for DDRT Only

	Hazards ratio (US resident vs. Undocumented Immigrants)			
Unadjusted	Unadjusted	HR*	95% CI	P
		2.84	[1.11,7.22]	0.02
Adjusted	Adjusted variables	HR*	95% CI	P
	**Demographics**			
	Age	2.53	[0.97,6.59]	0.06
	Race (AA)	2.94	[0.95-9.07]	0.06
	Ethnicity	2.23	[0.84,5.93]	0.11
	Obesity	2.83	[1.11,7.21]	0.03
	**Comorbidities**			
	CAD	3.13	[1.22,8.00]	0.02
	DM	2.71	[1.05,6.96]	0.04
	HTN	2.82	[1.11,7.19]	0.03
	Stroke	2.75	[1.08,7.02]	0.03
	HF	2.83	[1.11,7.22]	0.03
	Cancer	2.75	[1.08,7.03]	0.03
	**Transplant Characteristics**			
	CIT (/5 hour)	2.9	[1.14,7.4]	0.03
	CMV High risk	2.74	[1.07,7.02]	0.04
	DCD	2.75	[1.07,7.04]	0.03
	Dialysis duration	2.88	[1.13,7.33]	0.03
	DGF	2.28	[0.89,5.85]	0.09
	High PRA	2.85	[1.12,7.25]	0.03
	HLA	2.74	[1.07,6.99]	0.04
	Simulect Induction	2.81	[1.1,7.16]	0.03
	0MM	2.73	[1.06,6.99]	0.04
	**Donor Characteristics**			
	Age	2.79	[1.09,7.1]	0.02
	Dm	2.87	[1.12,7.34]	0.03
	Ethnicity	2.79	[1.1,7.1]	0.03
	Gender	2.83	[1.11,7.21]	0.03
	HTN	3.05	[1.19,7.82]	0.02

© 2023 Eguchi N et al. *JAMA Network Open.*

	Serum Cr	2.89	[1.13,7.37]	0.03

0MM, 0-human leukocyte antigen mismatch; AA, African American; CAD, coronary artery disease; CI, Confidence interval; CIT, cold ischemic time; CMV, cytomegalovirus; Cr, creatinine; DDKT, deceased donor kidney transplant; DGF, delayed graft function; DM, diabetes mellitus; HF, heart failure; HLA, human leukocyte antigen; HR, hazard ratio; HTN, hypertension; LDKT, living donor kidney transplant; PRA, panel reactive antibody; UI, undocumented immigrants; UR, US residents
* UI is a reference

© 2023 Eguchi N et al. *JAMA Network Open.*

eTable 3. Dialysis After Graft Loss (DAGL) for All Transplant

	Hazards ratio (US resident vs. Undocumented Immigrants)			
Unadjusted	Unadjusted	HR*	95% CI	P
		2.27	[0.78,6.56]	0.13
Adjusted	Adjusted variables	HR*	95% CI	P
	**Demographics**			
	Age	2.52	[0.84,7.5]	0.10
	Race (AA)	2.94	[0.95,9.07]	0.06
	Ethnicity	2.52	[0.84,7.53]	0.10
	Obesity	2.16	[0.75,6.26]	0.16
	**Comorbidities**			
	CAD	2.31	[0.80,6.7]	0.12
	DM	2.24	[0.76,6.56]	0.14
	HTN	2.250	[0.78,6.53]	0.13
	Stroke	2.33	[0.80,6.77]	0.12
	HF	2.22	[0.77,6.45]	0.14
	Cancer	2.1	[0.72,6.14]	0.17
	**Transplant Characteristics**			
	CIT (/5 hour)	2.32	[0.80,6.71]	0.12
	CMV High risk	2.23	[0.77,6.48]	0.14
	Dialysis duration	2.27	[0.78,6.56]	0.13
	DGF	1.78	[0.61,5.23]	0.29
	High PRA	2.27	[0.78,6.58]	0.13
	HLA	2.23	[0.77,6.49]	0.14
	Living Donor	2.28	[0.79,6.58]	0.13
	Simulect Induction	2.37	[0.82,6.87]	0.11
	0MM	2.22	[0.76,6.49]	0.14
	**Donor Characteristics**			
	Age	2.29	[0.79,6.64]	0.13
	Dm	2.37	[0.8,6.77]	0.12
	Ethnicity	2.17	[0.74,6.41]	0.16

© 2023 Eguchi N et al. *JAMA Network Open.*

	Gender	2.13	[0.73,6.2]	0.17
	HTN	2.40	[0.83,6.99]	0.11
	Serum Cr	2.35	[0.81,6.8]	0.12

0MM, 0-human leukocyte antigen mismatch; AA, African American; CAD, coronary artery disease; CI, Confidence interval; CIT, cold ischemic time; CMV, cytomegalovirus; Cr, creatinine; DDKT, deceased donor kidney transplant; DGF, delayed graft function; DM, diabetes mellitus; HF, heart failure; HLA, human leukocyte antigen; HR, hazard ratio; HTN, hypertension; LDKT, living donor kidney transplant; PRA, panel reactive antibody; UI, undocumented immigrants; UR, US residents
* UI is a reference

© 2023 Eguchi N et al. *JAMA Network Open.*

eTable 4. DAGL for DDRT Only

	Hazards ratio (US resident vs. Undocumented Immigrants)			
Unadjusted	Unadjusted	HR*	95% CI	P
		2.13	[0.62,7.29]	0.23
Adjusted	Adjusted variables	HR*	95% CI	P
	**Demographics**			
	Age	2.33	[0.67,8.16]	0.19
	Race (AA)	2.23	[0.62,8.12]	0.22
	Ethnicity	2.06	[0.58,7.34]	0.27
	Obesity	2.16	[0.63,7.40]	0.22
	**Comorbidities**			
	CAD	2.4	[0.7,8.27]	0.17
	DM	2.11	[0.61,7.34]	0.34
	HTN	2.14	[0.62,7.3]	0.23
	HF	2.09	[0.61,7.17]	0.24
	Cancer	1.99	[0.58,6.87]	0.28
	**Transplant Characteristics**			
	CIT (/5 hour)	2.28	[0.67,7.84]	0.19
	CMV High risk	2.08	[0.6,7.15]	0.25
	DCD	1.85	[0.53,6.44]	0.33
	Dialysis duration	2.17	[0.63,7.41]	0.22
	DGF	1.47	[0.42,5.14]	0.55
	High PRA	2.18	[0.64,7.45]	0.21
	HLA	2.13	[0.62,7.32]	0.23
	Simulect Induction	2.09	[0.61,7.15]	0.24
	0MM	3.98	[0.93,17]	0.05
	**Donor Characteristics**			
	Age	2.17	[0.63,7.42]	0.22
	Dm	2.13	[0.62,7.32]	0.23
	Ethnicity	2.04	[0.60,6.98]	0.26
	Gender	2.09	[0.61,7.16]	0.24
	HTN	2.30	[0.66,7.94]	0.19
	Serum Cr	2.24	[0.66,7.69]	0.20

© 2023 Eguchi N et al. *JAMA Network Open.*

0MM, 0-human leukocyte antigen mismatch; AA, African American; CAD, coronary artery disease; CI, Confidence interval; CIT, cold ischemic time; CMV, cytomegalovirus; Cr, creatinine; DDKT, deceased donor kidney transplant; DGF, delayed graft function; DM, diabetes mellitus; HF, heart failure; HLA, human leukocyte antigen; HR, hazard ratio; HTN, hypertension; LDKT, living donor kidney transplant; PRA, panel reactive antibody; UI, undocumented immigrants; UR, US residents
* UI is a reference

© 2023 Eguchi N et al. *JAMA Network Open.*

eTable 5. All-Cause Mortality for All Transplants

	Hazards ratio (US resident vs. Undocumented Immigrants)			
Unadjusted	Unadjusted	HR*	95% CI	P
		4.43	[1.05,18.69]	0.0424
Adjusted	Adjusted variables	HR*	95% CI	P
	**Demographics**			
	Age	2.57	[0.59,11.23]	0.21
	Race (AA)	3.62	[0.28,0.83]	0.087
	Ethnicity	3.28	[0.74,14.54]	0.12
	Obesity	4.28	[0.73,1.98]	0.048
	**Comorbidities**			
	CAD	4.51	[1.07,19.03]	0.04
	DM	3.87	[0.91,16.43]	0.07
	HTN	4.28	[1.02,18.07]	<0.05
	Stroke	4.11	[0.97,17.39]	0.06
	HF	4.44	[1.05,18.71]	0.04
	Cancer	4.33	[1.02,18.28]	<0.05
	**Transplant Characteristics**			
	CIT (/5 hour)	4.73	[1.12, 20]	0.03
	CMV High risk	4.21	[1, 17.83]	0.05
	Dialysis duration	4.43	[1.05,18.69]	0.04
	DGF	4.10	[0.97,17.32]	0.06
	High PRA	4.46	[1.06,18.79]	0.04
	HLA	4.30	[1.02,18.16]	<0.05
	Living Donor	4.75	[1.13,20.04]	0.03
	Simulect Induction	4.42	[1.05,18.66]	0.04
	0MM	4.41	[1.04,18.69]	0.04
	**Donor Characteristics**			
	Age	4.27	[1.01,18.03]	<0.05
	Dm	4.65	[1.09,19.75]	0.04
	Ethnicity	4.63	[1.09.19.61]	0.04
	Gender	4.66	[1.11,19.67]	0.04
	HTN	4.99	[1.17,21.26]	0.03

© 2023 Eguchi N et al. *JAMA Network Open.*

	Serum Cr	4.41	[1.05,18.57]	0.04

0MM, 0-human leukocyte antigen mismatch; AA, African American; CAD, coronary artery disease; CI, Confidence interval; CIT, cold ischemic time; CMV, cytomegalovirus; Cr, creatinine; DDKT, deceased donor kidney transplant; DGF, delayed graft function; DM, diabetes mellitus; HF, heart failure; HLA, human leukocyte antigen; HR, hazard ratio; HTN, hypertension; LDKT, living donor kidney transplant; PRA, panel reactive antibody; UI, undocumented immigrants; UR, US residents
* UI is a reference

© 2023 Eguchi N et al. *JAMA Network Open.*

eTable 6. All-Cause Mortality for DDRT Only

	Hazards ratio (US resident vs. Undocumented Immigrants)			
Unadjusted	Unadjusted	HR*	95% CI	P
		4.21	[0.99, 17.91]	0.05
Adjusted	Adjusted variables	HR*	95% CI	P
	**Demographics**			
	Age	2.45	[1.02,5.9]	0.04
	Race (AA)	3.46	[0.79,15.21]	0.1
	Ethnicity	2.66	[0.58,12.14]	0.21
	Obesity	4.15	[0.97,17.69]	0.05
	**Comorbidities**			
	CAD	5.59	[1.07,19.65]	0.04
	DM	3.75	[0.87,16.11]	0.08
	HTN	4.12	[0.97,17.56]	0.05
	Stroke	3.87	[0.91,16.56]	0.07
	HF	4.21	[0.99,17.91]	0.05
	Cancer	4.06	[0.95,17.36]	0.05
	**Transplant Characteristics**			
	CIT (/5 hour)	4.19	[0.98,17.87]	0.05
	CMV High risk	3.99	[0.93,17.04]	0.06
	DCD	1.85	[0.53,6.44]	0.33
	Dialysis duration	4.24	[1,18.06]	0.05
	DGF	3.68	[0.89,16.24]	0.07
	High PRA	4.33	[0.99,1796]	0.05
	HLA	3.95	[0.93,16.84]	0.06
	Simulect Induction	4.17	[0.98,17.81]	0.05
	0MM	3.98	[0.93,17]	0.05
	**Donor Characteristics**			
	Age	4.03	[0.95,17.19]	0.06
	Dm	4.31	[1,18.54]	0.049
	Ethnicity	4.17	[0.98,17.74]	0.05
	Gender	4.19	[0.98,17.86]	0.05

© 2023 Eguchi N et al. *JAMA Network Open.*

	HTN	4.53	[1.06,19.45]	0.04
	Serum Cr	4.15	[0.97,17.68]	0.05

0MM, 0-human leukocyte antigen mismatch; AA, African American; CAD, coronary artery disease; CI, Confidence interval; CIT, cold ischemic time; CMV, cytomegalovirus; Cr, creatinine; DDKT, deceased donor kidney transplant; DGF, delayed graft function; DM, diabetes mellitus; HF, heart failure; HLA, human leukocyte antigen; HR, hazard ratio; HTN, hypertension; LDKT, living donor kidney transplant; PRA, panel reactive antibody; UI, undocumented immigrants; UR, US residents
* UI is a reference

© 2023 Eguchi N et al. *JAMA Network Open.*

eTable 7. Rejection for All Transplant

	Hazards ratio (US resident vs. Undocumented Immigrants)			
Unadjusted	Unadjusted	HR*	95% CI	P
		1.44	[0.69,2.99]	0.33
Adjusted	Adjusted variables	HR*	95% CI	P
	**Demographics**			
	Age	1.67	[0.79,3.53]	0.18
	Race (AA)	1.73	[0.81,3.71]	0.16
	Ethnicity	1.85	[0.87,3.91]	0.11
	Obesity	1.42	[0.68,2.95]	0.35
	**Comorbidities**			
	CAD	1.44	[0.69,2.99]	0.33
	DM	1.45	[0.69,3.03]	0.33
	HTN	1.48	[0.71,3.07]	0.30
	Stroke	1.43	[0.69,2.99]	0.34
	HF	1.46	[0.7, 3.04]	0.31
	Cancer	1.40	[0.69,2.91]	0.38
	**Transplant Characteristics**			
	CIT (/5 hour)	1.47	[0.71,3.06]	0.3
	CMV High risk	1.42	[0.68,2.97]	0.35
	Dialysis duration	1.44	[0.69,2.99]	0.33
	DGF	1.34	[0.64,2.79]	0.44
	High PRA	1.43	[0.69,2.98]	0.34
	HLA	1.50	[0.72,3.13]	0.28
	Living Donor	1.44	[0.69,3]	0.33
	Simulect Induction	1.46	[0.7,3.03]	0.32
	0MM	1.46	[0.7,3.05]	0.32
	**Donor Characteristics**			
	Age	1.43	[0.69,2.98]	0.34
	Dm	1.51	[0.72,3.16]	0.29
	Ethnicity	1.58	[0.75,3.32]	0.23

© 2023 Eguchi N et al. *JAMA Network Open.*

	Gender	1.39	[0.67,2.89]	0.38
	HTN	1.48	[0.71,3.1]	0.30
	Serum Cr	1.48	[0.71,3.08]	0.29

0MM, 0-human leukocyte antigen mismatch; AA, African American; CAD, coronary artery disease; CI, Confidence interval; CIT, cold ischemic time; CMV, cytomegalovirus; Cr, creatinine; DDKT, deceased donor kidney transplant; DGF, delayed graft function; DM, diabetes mellitus; HF, heart failure; HLA, human leukocyte antigen; HR, hazard ratio; HTN, hypertension; LDKT, living donor kidney transplant; PRA, panel reactive antibody; UI, undocumented immigrants; UR, US residents
* UI is a reference

© 2023 Eguchi N et al. *JAMA Network Open.*

eTable 8. Rejection for DDRT Only

	Hazards ratio (US resident vs. Undocumented Immigrants)			
Unadjusted	Unadjusted	HR*	95% CI	P
		1.06	[0.47,2.37]	0.90
Adjusted	Adjusted variables	HR*	95% CI	P
	**Demographics**			
	Age	1.22	[0.53,2.77]	0.64
	Race (AA)	1.14	[0.49,2.66]	0.77
	Ethnicity	1.23	[0.54,2.82]	0.62
	Obesity	1.05	[0.47,2.36]	0.91
	**Comorbidities**			
	CAD	1.05	[0.47,2.36]	0.91
	DM	1.05	[0.47,2.39]	0.9
	HTN	1.09	[0.49,2.46]	0.83
	Stroke	1.06	[0.47,2.38]	0.9
	HF	1.06	[0.47,2.38]	0.89
	Cancer	1.06	[0.47,2.4]	0.88
	**Transplant Characteristics**			
	CIT (/5 hour)	1.11	[0.49,2.5]	0.81
	CMV High risk	1.04	[0.46,2.34]	0.93
	DCD	1.02	[0.45,2.3]	0.97
	Dialysis duration	1.06	[0.47,2.38]	0.9
	DGF	0.89	[0.39,2.03]	0.78
	High PRA	1.05	[0.47,2.36]	0.9
	HLA	1.15	[0.51,2.61]	0.73
	Simulect Induction	1.09	[0.48,2.47]	0.83
	0MM	1.07	[0.47,2.42]	0.88
	**Donor Characteristics**			
	Age	1.05	[0.47,2.37]	0.9

© 2023 Eguchi N et al. *JAMA Network Open.*

	Dm	1.11	[0.49,2.54]	0.8
	Ethnicity	1.05	[0.47,2.37]	0.9
	Gender	1.06	[0.47,2.39]	0.89
	HTN	1.09	[0.48,2.47]	0.83
	Serum Cr	1.11	[0.49,2.5]	0.8

0MM, 0-human leukocyte antigen mismatch; AA, African American; CAD, coronary artery disease; CI, Confidence interval; CIT, cold ischemic time; CMV, cytomegalovirus; Cr, creatinine; DDKT, deceased donor kidney transplant; DGF, delayed graft function; DM, diabetes mellitus; HF, heart failure; HLA, human leukocyte antigen; HR, hazard ratio; HTN, hypertension; LDKT, living donor kidney transplant; PRA, panel reactive antibody; UI, undocumented immigrants; UR, US residents
* UI is a reference

© 2023 Eguchi N et al. *JAMA Network Open.*

A)

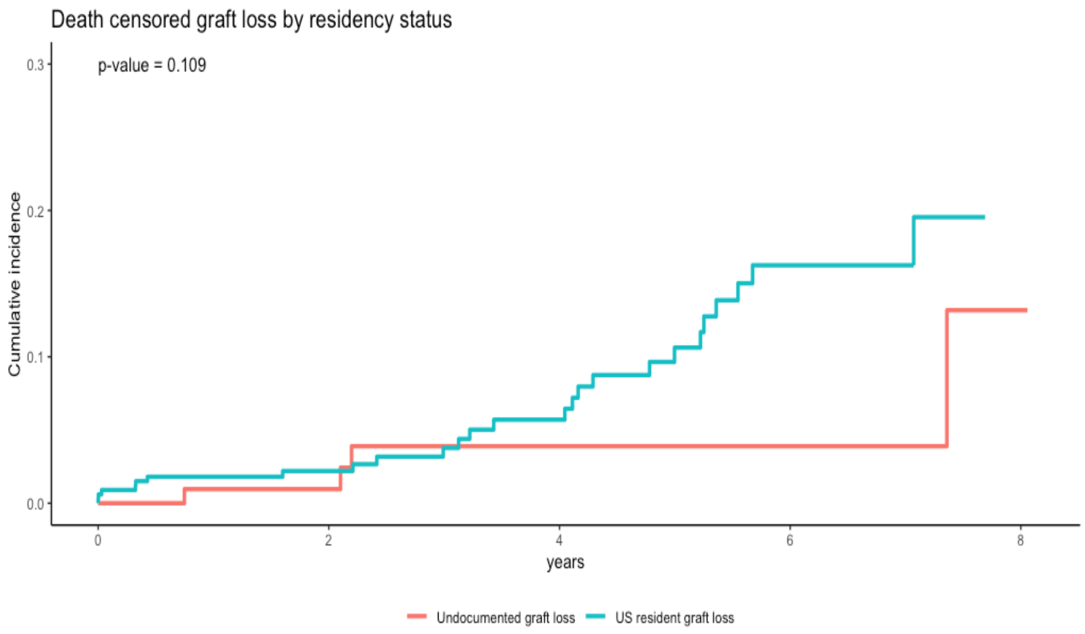

B)

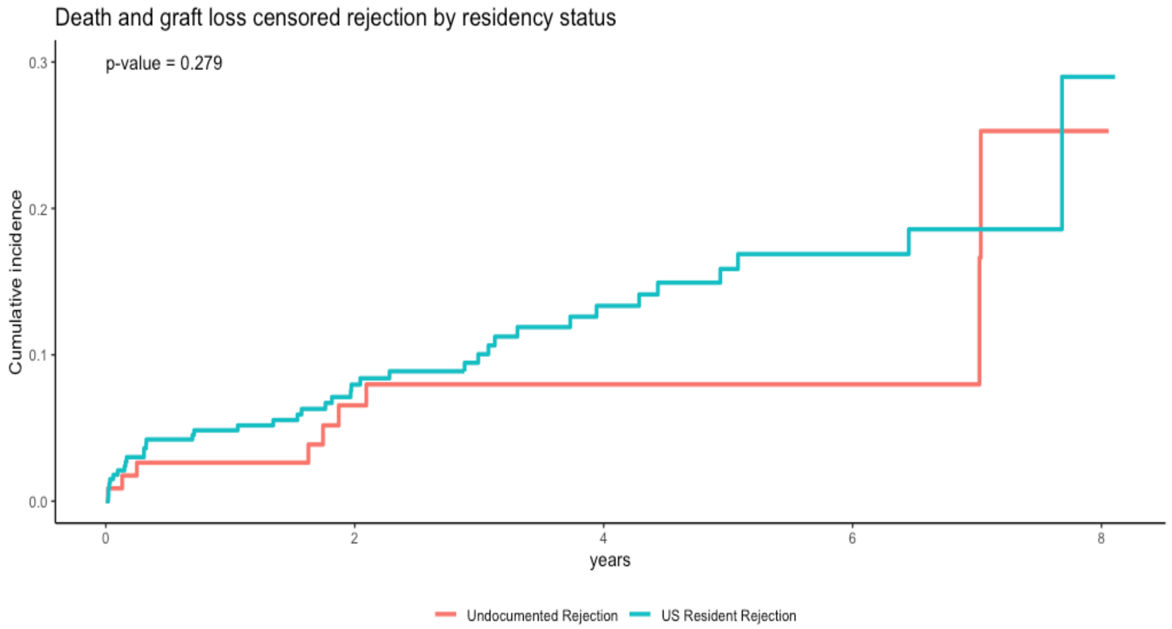

eFigure 1. Cumulative Incidence Curve for A) Death Censored Graft Loss B) Death and Graft Loss Censored Rejection by Residency Status

© 2023 Eguchi N et al. *JAMA Network Open.*

eFigure 2. Conceptual Framework

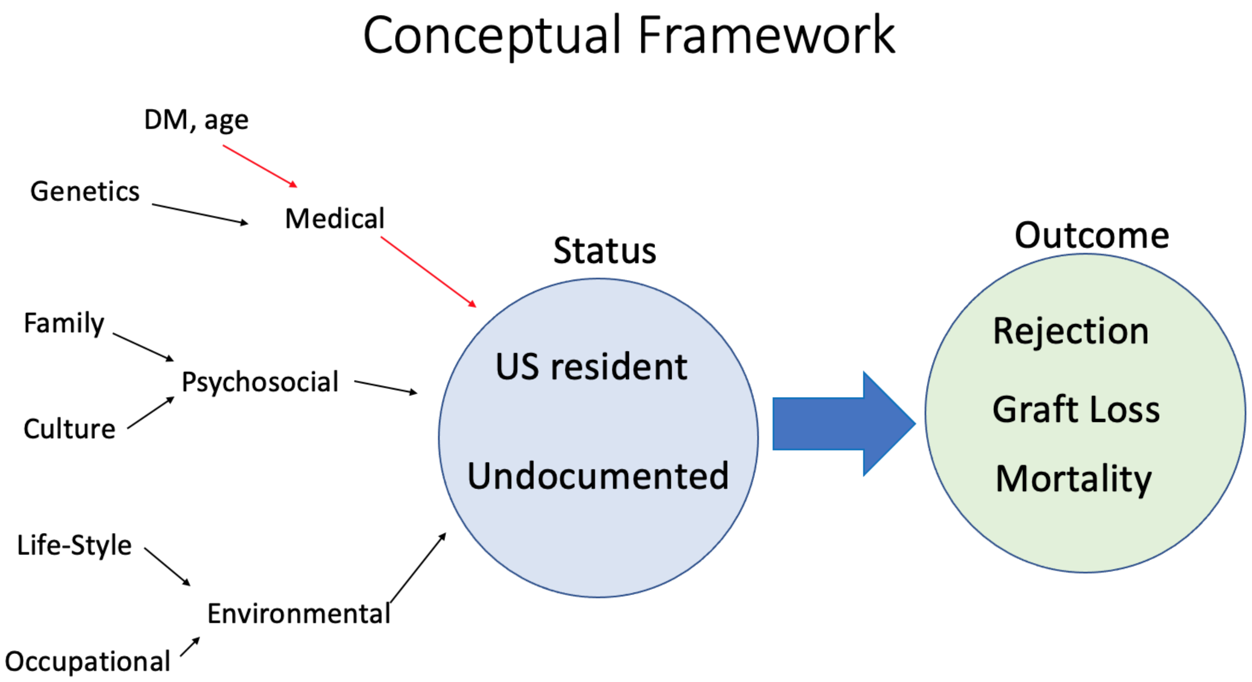

© 2023 Eguchi N et al. *JAMA Network Open.*
