# Supplementary material for: Outcomes Among Undocumented Immigrant Kidney Transplant Recipients in California
Source: JAMA Netw Open. 2023 Feb 13;6(2):e2254660. doi: 10.1001/jamanetworkopen.2022.54660 (PMC9926318; doi:10.1001/jamanetworkopen.2022.54660)
Supplement: Supplement 2. — Data Sharing Statement [file jamanetwopen-e2254660-s002.pdf]

Data Sharing Statement

Eguchi. Outcomes Among Undocumented Immigrant Kidney Transplant Recipients in California. *JAMA Netw Open*. Published February 13, 2023.
doi:10.1001/jamanetworkopen.2022.54660

Data
Data available: Yes
Data types: Deidentified participant data
How to access data: Hirohito Ichii hichii@uci.edu
When available: With publication

Supporting Documents
Document types: None

Additional Information
Who can access the data: researchers whose proposed use of the data has been approved
Types of analyses: for any purpose
Mechanisms of data availability: after approval of a proposal
